# Prediction of Acquired Antimicrobial Resistance for Multiple Bacterial Species Using Neural Networks

**DOI:** 10.1128/mSystems.00774-19

**Published:** 2020-01-21

**Authors:** D. Aytan-Aktug, P. T. L. C. Clausen, V. Bortolaia, F. M. Aarestrup, O. Lund

**Affiliations:** a National Food Institute, Technical University of Denmark, Kongens Lyngby, Denmark; Marquette University

**Keywords:** AMR, antimicrobial resistance, machine learning, neural networks

## Abstract

Machine learning has proven to be a powerful method to predict antimicrobial resistance (AMR) without using prior knowledge for selected bacterial species-antimicrobial combinations. To date, only species-specific machine learning models have been developed, and to the best of our knowledge, the inclusion of information from multiple species has not been attempted. The aim of this study was to determine the feasibility of including information from multiple bacterial species to predict AMR for an individual species, since this may make it easier to train and update resistance predictions for multiple species and may lead to improved predictions. Whole-genome sequence data and susceptibility profiles from 3,528 Mycobacterium tuberculosis, 1,694 Escherichia coli, 658 Salmonella enterica, and 1,236 Staphylococcus aureus isolates were included. We developed machine learning models trained by the features of the PointFinder and ResFinder programs detected to predict binary (susceptible/resistant) AMR profiles. We tested four feature representation methods to determine the most efficient way for introducing features into the models. When training the model only on the Mycobacterium tuberculosis isolates, high prediction performances were obtained for the six AMR profiles included. By adding information on ciprofloxacin from the additional 3,588 isolates, there was no reduction in performance for the other antimicrobials but an increased performance for ciprofloxacin AMR profile prediction for Mycobacterium tuberculosis and Escherichia coli. In conclusion, the species-independent models can predict multi-AMR profiles for multiple species without losing any robustness.

**IMPORTANCE** Machine learning is a proven method to predict AMR; however, the performance of any machine learning model depends on the quality of the input data. Therefore, we evaluated different methods of representing information about mutations as well as mobilizable genes, so that the information can serve as input for a robust model. We combined data from multiple bacterial species in order to develop species-independent machine learning models that can predict resistance profiles for multiple antimicrobials and species with high performance.

## Abstract (precis)

Machine learning is a proven method to predict AMR; however, the performance of any machine learning model depends on the quality of the input data. Therefore, we evaluated different methods of representing information about mutations as well as mobilizable genes, so that the information can serve as input for a robust model. We combined data from multiple bacterial species in order to develop species-independent machine learning models that can predict resistance profiles for multiple antimicrobials and species with high performance.

## INTRODUCTION

Antimicrobials have been used for infectious disease treatment for decades. Bacteria may develop antimicrobial resistance (AMR), making them insensitive to certain antibiotic treatments (1). AMR in bacteria is mainly mediated by the acquisition of chromosomal mutations and/or the horizontal acquisition of mobilizable genes. To date, many patients with infectious diseases have been prescribed inappropriate antibiotics, leading to increased mortality and health care costs due to AMR (2). It is therefore vital to accurately determine the AMR profiles of microorganisms causing infections (3).

Species identification and antimicrobial susceptibility testing have traditionally been performed using conventional testing of the bacterial phenotype. However, with the increasing availability of next-generation sequencing for routine diagnostics, it has been suggested that species identification and antimicrobial susceptibility testing might be performed in a single analysis based on the genomic sequence (4).

A number of bioinformatics tools, including the ResFinder (18) and PointFinder (6) programs, that predict AMR from DNA sequence data using prior knowledge have been developed and made available for the global research and diagnostic communities. PointFinder detects chromosomal mutations for a few selected bacterial species, while ResFinder solely detects mobilizable genes. Since 2018, these two prediction tools have been merged and are available on the ResFinder (version 3.0) web server. However, it has been shown that such tools are highly dependent on the literature, as they provide accurate predictions only for the well-studied AMR mechanisms (8).

Machine learning represents an alternative method to predict AMR from sequence data without the need for prior knowledge of chromosomal mutations and mobilizable genes (8). Many machine learning methods can take into consideration the effects of combinations of mutations and/or mobilizable genes. To date, several studies have been able to predict AMR profiles for various bacterial species and antibiotic combinations using different machine learning algorithms (2, 8–11). The major variation between the studies is how the bacterial genomes are translated into features that are then used as input to the machine learning methods. These studies can roughly be divided into methods that utilize *k*-mers, single nucleotide polymorphisms (SNPs), AMR genes, and whole-genome sequence (WGS) data as features (2, 12, 13). The success of machine learning is not limited by the available literature or whether the organism is known or not. In contrast to the literature-based methods, machine learning may also be capable of discovering new resistance mechanisms.

Here we present machine learning models trained with chromosomal mutations and mobilizable genes. As the data representation methods play an important role in obtaining an accurate prediction of AMR profiles, we evaluated the effect of using different data representations. We demonstrate that performances improved over those of Point-/ResFinder can be obtained using the mutations and genes identified by Point-/ResFinder as input, but without parsing the information input into the machine learning methods about which mutations and genes are known to be associated with AMR. Importantly, we demonstrate that information from one species might improve the predictions for other species. Thus, we demonstrate that it is possible to develop robust multioutput and species-independent models for AMR typing to also eventually cover species for which no prior knowledge is available.

## RESULTS

Genetic variations that may mediate AMR were detected using the ResFinder and PointFinder programs. These genetic factors were then presented to the machine learning methods by the use of various representations. These representations included binary representation, scored representation, amino acid representation, nucleotide representation, and a combination of the binary and scored representations, as described in Materials and Methods. In order to compare the robustness of these representation methods, the random forest model was applied to the Escherichia coli and Mycobacterium tuberculosis isolates to predict ciprofloxacin and rifampin resistance profiles, respectively. The results are provided in Table 1. Due to the 5-fold cross-validation method applied, all of the results obtained with the validation data are the averages of the values obtained with the five different machine learning models.

**TABLE 1:** Application of random forest models to the differently represented E. coli and M. tuberculosis data sets^*a*^

Species (no. of isolates) and data representation method (no. of features)	AUC	
	Validation data	Test data
E. coli (1,694)		
Binary representation (1,119)	0.98 ± 0.01	0.97
Scored representation (2,167)	0.98 ± 0.01	0.97
Scored + binary representation (4,219)	0.98 ± 0.01	0.98
Amino acid representation (52,199)	0.98 ± 0.01	0.97
Nucleotide representation (14,483)	0.98 ± 0.02	0.97
M. tuberculosis (1,785)		
Binary representation (6,735)	0.94 ± 0.04	0.92
Scored representation (11,120)	0.94 ± 0.04	0.92
Scored + binary representation (21,975)	0.94 ± 0.04	0.92
Amino acid representation (261,085)	0.93 ± 0.04	0.92
Nucleotide representation (87,205)	0.93 ± 0.04	0.92

a For the performances with E. coli, the model was trained and validated with 1,422 isolates and tested with 272 isolates. For the performances with M. tuberculosis, the model was trained and validated with 992 isolates and tested with 793 isolates. All of these M. tuberculosis isolates had complete resistance profiles. AUC, area under the curve.

For M. tuberculosis and E. coli, predictions based on the different representations performed equally well (*P* value 0.5 > significance threshold 0.05), and the best performance was observed for the combination method with E. coli. In the further analyses, use of the combination of the binary and scored representation methods was chosen, as this combination maintains the simplicity of the binary model in combination with the preciseness of the scoring model, while it has fewer features than the amino acid and nucleotide representations.

### Single output versus multiple outputs.

We then tested the prediction power of individual prediction models for each antimicrobial against an aggregated multioutput model. The random forest and neural network models were applied to predict all the AMR profiles for the M. tuberculosis isolates both together and separately, as shown in Table 2. The single- and multioutput model performances were compared by a paired *t* test. The *P* value for both of the test results was 0.374 using random forests and neural networks. Therefore, the null hypothesis that there is no difference in the performances between the single- and multioutput models was accepted for the 0.05 significance threshold.

**TABLE 2:** Application of random forest and neural network models to M. tuberculosis data^*a*^

Model and antimicrobial	AUC			
	Single output		Multiple outputs	
	Validation data	Test data	Validation data	Test data
Random forests (1,785 isolates)				
Rifampin	0.94 ± 0.04	0.92	0.92 ± 0.06	0.92
Isoniazid	0.91 ± 0.03	0.93	0.91 ± 0.03	0.93
Streptomycin	0.89 ± 0.05	0.91	0.88 ± 0.07	0.90
Ethambutol	0.89 ± 0.09	0.80	0.86 ± 0.08	0.80
Pyrazinamide	0.82 ± 0.06	0.79	0.83 ± 0.09	0.79
Neural networks (3,528 isolates)				
Rifampin	0.95 ± 0.04	0.94	0.94 ± 0.04	0.94
Isoniazid	0.90 ± 0.02	0.94	0.88 ± 0.04	0.94
Streptomycin	0.86 ± 0.05	0.88	0.86 ± 0.07	0.87
Ethambutol	0.94 ± 0.06	0.87	0.93 ± 0.06	0.87
Pyrazinamide	0.86 ± 0.05	0.83	0.83 ± 0.08	0.83

a The genetic variations are represented by the combination of the binary and scored representations. The model with random forests was trained and validated with 992 isolates and tested with 793 isolates, and the model with neural networks was trained and validated with 2,293 isolates and tested with 1,235 isolates. The M. tuberculosis data used in the random forest model included only the complete resistance profiles, whereas the data used in the neural network model did not. This explains why more isolates were included in the neural network model than in the random forest model. As the number of M. tuberculosis isolates having AMR profiles for all antimicrobials was only 92, ciprofloxacin was discarded for these models.

The random forest and neural network models performed equally well, as shown in Table 2. In order to handle the missing outputs, we imputed missing output values for the random forest model. The missing values were replaced by the predictions obtained by a random forest model that was trained only with the isolates having no missing values. The imputed data were used to predict resistance profiles, whereas the imputed data for the isolates were not considered in the model assessment process. Because of the imputation process, we continued with the artificial neural networks.

Additionally, in order to gain more insight into the predictions made by the random forest and neural network models, the first 20 and 50 most important features per species are provided in Tables S3 and S4 in the supplemental material for the random forest and neural network models, respectively, and Data Sets S2 and S3 for the random forest and neural network models, respectively.

10.1128/mSystems.00774-19.5 TABLE S3 The first 20 most important features demonstrated were generated by the random forest models. Each feature listed in the top 20 most important feature list at least three times in the 5-fold cross-validation loop. The features overlapping the Point-/ResFinder databases are written in bold. S. aureus results were not comparable because the PointFinder database did not include any information regarding ciprofloxacin at the time of testing. For each mutation, the order is GeneName_Position_WildType. For each mobilizable gene, the order is GeneName_VariantInResFinder_AccessionNumber. Del, deletion. Download Table S3, DOCX file, 0.01 MB. Copyright © 2020 Aytan-Aktug et al. 2020 Aytan-Aktug et al. This content is distributed under the terms of the Creative Commons Attribution 4.0 International license.

### Single species versus multiple species.

The next comparison made in the study was between the performances of the models for single and multiple species. The data set that included multiple species was generated by merging all the single species into one data set using the first input merging method (see Materials and Methods). The performances of both models are shown as area-under-the-curve (AUC) values in Table 3, and the difference between the model performances was tested using a paired *t* test. For the test results, the *P* value was 0.488. The null hypothesis that there is no difference in performances between the single- and multispecies models was accepted at the 0.05 significance threshold. In the assembly process, one of the Staphylococcus aureus isolates (Pathosystems Resource Integration Center [PATRIC] accession number 1280.9410) and eight Salmonella enterica isolates (PATRIC accession numbers 340190.17, 340190.19, 340190.21, 90105.22, 90105.18, 90105.19, 340190.7, and 340190.20) failed. These failed isolates were not included in the models.

**TABLE 3:** Application of neural network model to single species data and merged species data^*a*^

Antimicrobial	AUC for the following species (no. of isolates):									
	E. coli (1,694)		M. tuberculosis (3,528)		S. enterica (658)		S. aureus (1,236)		E. coli, M. tuberculosis, S. enterica, and S. aureus	
	Validation data	Test data	Validation data	Test data	Validation data	Test data	Validation data	Test data	Validation data	Test data
Rifampin	NA	NA	0.94 ± 0.04	0.94	NA	NA	NA	NA	0.94 ± 0.04	0.95
Isoniazid	NA	NA	0.88 ± 0.04	0.94	NA	NA	NA	NA	0.93 ± 0.03	0.94
Streptomycin	NA	NA	0.89 ± 0.06	0.87	NA	NA	NA	NA	0.89 ± 0.06	0.89
Ethambutol	NA	NA	0.93 ± 0.06	0.87	NA	NA	NA	NA	0.91 ± 0.05	0.92
Pyrazinamide	NA	NA	0.82 ± 0.11	0.83	NA	NA	NA	NA	0.85 ± 0.04	0.87
Ciprofloxacin	0.99 ± 0.01	0.97	0.90 ± 0.12	0.97	0.75 ± 0.15	0.85	0.98 ± 0.01	0.99	0.98 ± 0.01	0.97

a The results for M. tuberculosis were slightly different from the results shown in Table 2, as the output dimension was changed. NA, the result is not available.

### Discrete databases versus concatenated database.

Two different ways of generating a multiple-species data set were tested in this study. In method 1 (see Materials and Methods), the sequence of each species was aligned only to its own reference sequence.

In method 2, the sequence of each species was aligned to the sequences in a concatenated reference database, and the sequences of 197 isolates failed to align.

**FIG 1:**
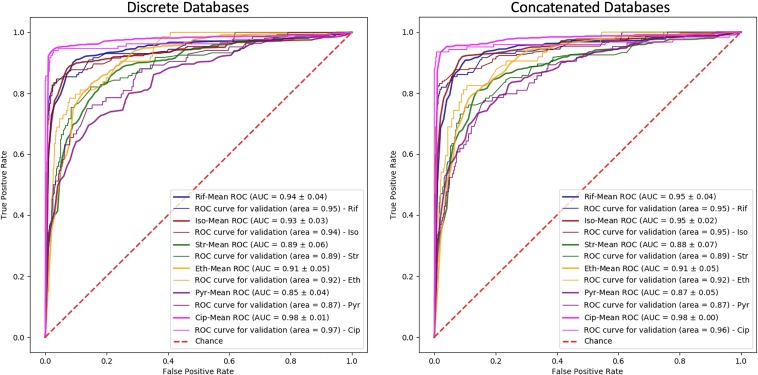
The ROC curves are shown for the models trained with the multispecies data sets. (Left) The model was trained with the data aligned to the discrete databases; (right) the model was trained with the data aligned to the concatenated databases. Rif, rifampin; Iso, isoniazid; Str, streptomycin; Eth, ethambutol; Pyr, pyrazinamide; Cip, ciprofloxacin.

**FIG 2:**
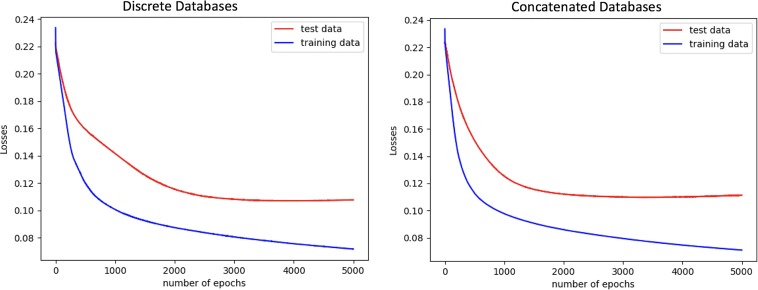
The model training and validation data loss plots are shown. The models were trained with the multispecies data sets generated by aligning to the discrete (left) and concatenated (right) databases. The plots show that the models do not indicate overfitting when iterated 5,000 times.

The one-hidden-layer neural network model was applied to the multispecies data sets, and the model performances are shown in Table 4. The model receiver operating characteristic (ROC) curves and loss plots, along with the performances, are shown in Fig. 1 and 2, respectively.

**TABLE 4:** Application of neural network models to multispecies data sets, which were produced by the different methods

Antimicrobial	AUC for E. coli, M. tuberculosis, S. enterica, and S. aureus			
	Discrete databases		Concatenated databases	
	Validation data	Test data	Validation data	Test data
Rifampin	0.94 ± 0.04	0.95	0.95 ± 0.04	0.95
Isoniazid	0.93 ± 0.03	0.94	0.95 ± 0.02	0.95
Streptomycin	0.89 ± 0.06	0.89	0.88 ± 0.07	0.89
Ethambutol	0.91 ± 0.05	0.92	0.91 ± 0.05	0.92
Pyrazinamide	0.85 ± 0.04	0.87	0.87 ± 0.05	0.87
Ciprofloxacin	0.98 ± 0.01	0.97	0.98 ± 0.00	0.96

According to the paired *t* test results, the *P* value for the test results was 0.404. The null hypothesis that there is no difference between the performances of the discrete and concatenated databases was accepted at the significance threshold of 0.05.

The one-hidden-layer neural network model performances for the multispecies data were compared with the multiple-hidden-layer neural network model performances, and no significant change was observed between the one-hidden-layer model and each individual multiple-hidden-layer models (*P* values ∈ [0.175, 0.363] > significance threshold 0.05). The performances of the multiple-hidden-layer neural network models with data from the concatenated database are shown in Fig. S1.

10.1128/mSystems.00774-19.1 FIG S1 The ROC curves are shown for the models trained with the multispecies data set. (a) The neural network model has three hidden layers, including 200, 100, and 200 hidden neurons, respectively. (b) The neural network model has two hidden layers, including 200 hidden neurons in each hidden layer. (c) The neural network model has two hidden layers, including 200 and 500 hidden neurons, respectively. (d) The neural network model has two hidden layers, including 500 200 hidden neurons, respectively. The multispecies data aligned to the concatenated databases. Download FIG S1, PDF file, 2.9 MB. Copyright © 2020 Aytan-Aktug et al. 2020 Aytan-Aktug et al. This content is distributed under the terms of the Creative Commons Attribution 4.0 International license.

In our theory, when the multispecies model was trained with a sufficient number of species, the model should be able to predict the AMR profiles for new species never before trained by the model. In this study, we attempted to show that with a simple experiment. We trained our one-hidden-layer neural network model with all of the species used in the study, which were E. coli, M. tuberculosis, S. enterica, and S. aureus, to teach the model ciprofloxacin resistance patterns. Afterwards, the model was tested with a completely new data set including data for 900 Klebsiella pneumoniae isolates and ciprofloxacin resistance profiles retrieved from the PATRIC database. All of the steps followed in the study were completed for this new data set, such as detecting mutations, insertion/deletions (indels), and mobilizable genes, using the Point-/ResFinder tools. However, the sequences in the new data set including data for K. pneumoniae isolates were aligned to those in the concatenated database, which does not include the sequence of the K. pneumoniae reference genome. As expected, the sequence of the whole K. pneumoniae genome did not align to the sequences in the concatenated database, and only part of it aligned to the E. coli and S. enterica reference gene sequences. The prediction of ciprofloxacin resistance for K. pneumoniae was close to random and is shown in Fig. S2.

10.1128/mSystems.00774-19.2 FIG S2 The ROC curve is shown for the model trained with the multispecies data sets. The model was tested with the K. pneumoniae isolates, which the model was never trained with. Download FIG S2, TIF file, 0.1 MB. Copyright © 2020 Aytan-Aktug et al. 2020 Aytan-Aktug et al. This content is distributed under the terms of the Creative Commons Attribution 4.0 International license.

### Point-/ResFinder versus machine learning.

The prediction performances of the machine learning models were compared with the prediction performances obtained with the Point-/ResFinder programs. The machine learning models used for the comparison were neural networks for all the species. All of the data sets were generated by combining the scored and binary representation methods. The performances were measured as the Matthews correlation coefficient (MCC) (14), shown in Table 5, because Point-/ResFinder can produce only a binary output. In addition, sensitivity, specificity, and F-1 scores are provided in Table S2. The probability threshold for the resistant isolates was set to 0.5 for the machine learning predictions.

**TABLE 5:** Point-/ResFinder results compared with the machine learning predictions^*a*^

Species-drug combination	MCC			
	Point-/ResFinder		Machine learning	
	Validation data	Test data	Validation data	Test data
E. coli-ciprofloxacin	0.71	0.70	0.96	0.94
M. tuberculosis-ciprofloxacin	0.91	0.71	0.62	0.78
M. tuberculosis-rifampin	0.83	0.87	0.72	0.81
M. tuberculosis-isoniazid	0.86	0.88	0.56	0.71
M. tuberculosis-streptomycin	0.61	0.78	0.39	0.58
M. tuberculosis-ethambutol	0.60	0.60	0.18	0.18
M. tuberculosis-pyrazinamide	0.60	0.60	0.38	0.28
S. enterica-ciprofloxacin	0.89	0.89	0.49	0.37
S. aureus-ciprofloxacin	NA	NA	0.97	0.95

a The PointFinder and ResFinder programs were not able to predict ciprofloxacin resistance for S. aureus because the database did not contain any prior knowledge on this at the time of testing. NA, the result is not available.

10.1128/mSystems.00774-19.4 TABLE S2 Sensitivity, specificity, and F-1 scores for machine learning and Point-/ResFinder predictions. The probability threshold for the resistant isolates is 0.5. Download Table S2, DOCX file, 0.01 MB. Copyright © 2020 Aytan-Aktug et al. 2020 Aytan-Aktug et al. This content is distributed under the terms of the Creative Commons Attribution 4.0 International license.

## DISCUSSION

The primary objective of this study was to test the feasibility of developing a multioutput and species-independent model for the prediction of AMR. This was accomplished by artificial neural networks trained with multispecies data. Furthermore, we also improved the Point-/ResFinder tools’ predictive capacity by predicting AMR profiles not encompassed by the databases through the use of machine learning models.

### Multispecies model.

To date, there are many bacterial species that have not been either annotated or very well studied, so little is known about them (15). Even in these cases, with no information available in the literature, the species-independent models presented here allow the prediction of resistance profiles regardless of the number of species in the data set. An approach similar to the one presented here may prove useful in directly predicting AMR from metagenomic samples. Metagenomic approaches have the advantages of avoiding culture bias and probably of having turnaround times shorter than those of classical microbiology methods (16). Moreover, by combining the species-independent model with the multioutput model, one can predict multiple resistance profiles for multiple species in a time-efficient manner.

On the other hand, our study missed a diverse data set including more species in order to reach accurate predictions for metagenomics studies. As shown in the experiment with K. pneumoniae, the model is not able to predict the AMR profile of a completely unknown species. Therefore, in any such future studies, the training data should be enriched with related species (e.g., same genus) to the unknown species.

### Data representation through Point-/ResFinder.

In contrast to other similar studies, this study did not rely solely on *k*-mer information as the input data type. Instead, we applied the PointFinder and ResFinder programs to detect the chromosomal mutations and mobilizable genes, respectively.

One drawback of this approach might be that the Point-/ResFinder tools restrict the features to a limited number of genes and thereby miss some information due to the lack of whole-genome information. Nevertheless, a recent study has shown that AMR gene information can provide enough information to the model (2). It has been proven by the performances of our models that the compulsory information buried in the core resistome is sufficient. Moreover, the models developed here overcome some of the limitations of the Point-/ResFinder tools. The robustness of Point-/ResFinder depends on the information provided in the literature and on the manual database curation. These databases do not include all existing chromosomal mutations and mobilizable genes mediating AMR, which are then rendered undetectable by these tools, but remain interpretable. Hereby, the machine learning models have the advantage of limiting the amount of prior knowledge and database curation needed to predict AMR. This was the case for the ciprofloxacin resistance profiles, which were predictable by the machine learning models for S. aureus, even though they were not included in PointFinder at the time of testing.

An advantage of using the genes in the PointFinder database instead of the whole genome is that the plasticity problem is overcome (17). Plasticity implies that reference genomes of the same bacterial species may be different. For this reason, we found the AMR genes in the PointFinder database to be more reliable, as they are manually curated after screening several reference genes (18).

Some of the features important for the random forest and neural network model predictions were not found to be associated with any of the resistance profiles provided by the Point-/ResFinder databases. This implies that machine learning might allow the discovery of new chromosomal mutations and mobilizable genes mediating AMR, which can be verified experimentally. Furthermore, some of the unassociated features might be the consequence of coresistance.

### Point-/ResFinder versus machine learning performances.

Regarding the second objective of the study, the machine learning prediction results were compared with the Point-/ResFinder results.

For E. coli, the machine learning method performed better than the Point-/ResFinder tools in predicting ciprofloxacin resistance. Point-/ResFinder predict AMR using the epidemiological cutoff values, whereas the machine learning models were fed the AMR profiles interpreted using clinical breakpoints. Therefore, the discordant performances might be the consequence of the gap between the epidemiological cutoff values and the clinical breakpoints. For instance, the *gyrA* (S83L) mutation without the presence of the *gyrA* (D87G) and *parC* (S80I) mutations causes only low-level ciprofloxacin resistance in E. coli and does not yield resistance according to clinical breakpoints (19). Thus, this single *gyrA* mutation causes only epidemiological resistance and not clinical resistance. Moreover, to overcome the inconsistency between the interpretive criteria for resistance, it might be more appropriate to predict minimum inhibitory concentration (MIC) values rather than binary resistance profiles.

For M. tuberculosis, the Point-/ResFinder tools performed significantly better than the machine learning models for predicting ethambutol and pyrazinamide resistance profiles (*P* value 0.006 < significance threshold 0.05). However, it has been discussed by previous studies (20, 21) that phenotypic drug susceptibility testing (DST) is unreliable for ethambutol and pyrazinamide. Due to the unreliability of DST, the poor machine learning performances might be the consequence of incorrectly profiled isolates; in that case, the prior knowledge used to build the Point-/ResFinder database might be biased.

For S. enterica, the machine learning model obtained one of the lowest performances according to the AUC, in which the AUC was far below that obtained with the Point-/ResFinder tools. S. enterica had the most imbalanced data set in the study: only 35 out of 658 isolates (5%) were resistant. In the S. enterica case, we observed that the random forest model provided a better performance than the neural network model. Without up-sampling, the random forest model reached AUCs of 0.87 ± 0.07 and 0.95 for the validation and test data sets, respectively, and the neural network model reached AUCs of 0.74 ± 0.15 and 0.87 for the validation and test data sets, respectively (these results are not shown in the Results section). The artificial neural network model is more complex than the random forest model and requires larger data sets for proper performance. The most probable reason for the low performance of the neural network model according to the AUC obtained is the limited number of resistant isolates in the data set. As discussed below, there was no significant difference between the performances of the neural network and random forest models with E. coli, 20% of isolates of which were resistant. This supports the idea that the neural network model for S. enterica requires more resistant isolates for more accurate predictions. However, these low performances were eliminated by learning through other species.

It was expected that the machine learning models would provide more accurate predictions than the Point-/ResFinder tools. In contrast, we observed that Point-/ResFinder outperformed the machine learning models in six out of eight cases. The antimicrobials tested in the models have been well studied, and most of the resistance mechanisms have been discovered so far. This implies that the Point-/ResFinder tools have sufficient prior knowledge to predict AMR profiles with few mistakes. For the less-studied AMR mechanisms, such as those conferring resistance to azithromycin and tigecycline, the machine learning models are expected to outperform Point-/ResFinder, as the advantage of the prior knowledge will be limited.

### Feature representation methods.

The data representation methods are supposed to play a crucial role in the machine learning methods. Ideally, the data should be informative and nonredundant in order to optimize the learning of the models. For this purpose, we tested different representation methods. The first method, binary representation, was the simplest representation method tested in this study. This method is considered simple because it does not convey information on which amino acid or nucleotide is the wild type or the mutant but merely contains information about the presence/absence of a mutation. The second method, scored representation, includes a more detailed representation of each identified SNP, but this extra information comes with the possibility of adding extra noise to the model as well. The third model, amino acid representation, simplifies the identification of which amino acid or nucleotide is responsible for the resistance profiles, as each of the possibilities is introduced into the data. The biggest disadvantage of this representation method is that it produces a large number of features, which has a tendency to decrease the signal-to-noise ratio for small data sets. The fourth method, nucleotide representation, might reduce the preciseness of the amino acid representation method. The reason for this is that the method includes only positions having nucleotide mutations, but it does not inform the model about the consequence of the amino acid changes. However, this method drops the feature expansion caused by the amino acid representation method. Despite these differences, we did not observe any significant difference in the results obtained between the data representation methods.

### Random forest model versus neural network model.

Each of the machine learning models applied in this study has different advantages. The random forest model is a simple ensemble model. The model has a limited number of hyperparameters and this makes it easier to tune. The random forest model achieved good performances for prediction of the AMR profiles for E. coli and M. tuberculosis for the different representation types. It was, however, challenging to deal with the missing outputs with the currently available implementations of the random forest model in the Scikit-learn Python package. We found the PyTorch Python package implementation of neural networks more convenient for handling missing values.

The artificial neural network model performs nonlinear mapping from the input layer to the output layer through the use of an extensive number of parameters which are tuned to allow flexibility. While this flexibility endures complex models, it is also a cause of overfitting problems. The model is capable of handling missing values and supports multioutput problems. In terms of model performance, we did not detect any significant difference between the artificial neural network and the random forest algorithms.

### Conclusion.

In this study, machine learning models were made eligible for performing multioutput and multispecies tasks without losing any prediction power. By introducing a new methodology for the Point-/ResFinder tools, the tool predictions are not particularly improved since Point-/ResFinder have the advantage of strong prior knowledge for the species-antimicrobial combinations tested. However, the models were capable of predicting the ciprofloxacin resistance profile for at least one species (S. aureus) not included in the Point-/ResFinder database. Further studies are needed to validate the multispecies predictions, including additional antimicrobial agents, bacterial isolates, and bacterial species, to capture the global AMR variation. This will pave the way for applying multitask machine learning models to metagenomic studies.

## MATERIALS AND METHODS

### Data sets.

The study covered four bacterial species: Mycobacterium tuberculosis, Escherichia coli, Salmonella enterica, and Staphylococcus aureus. Data for 3,528 M. tuberculosis isolates were obtained from the Relational Sequencing TB Data Platform at platform.reseqtb.org (22), and data for 1,694 E. coli, 658 S. enterica, and 1,236 S. aureus isolates were obtained from the Pathosystems Resource Integration Center (PATRIC) database (ftp://ftp.patricbrc.org/genomes_by_species/) (23). All the accession numbers are available in Data Set S1 in the supplemental material.

10.1128/mSystems.00774-19.7 DATA SET S1 Accession numbers for all isolates tested in this study. Download Data Set S1, XLSX file, 0.1 MB. Copyright © 2020 Aytan-Aktug et al. 2020 Aytan-Aktug et al. This content is distributed under the terms of the Creative Commons Attribution 4.0 International license.

All of the AMR profiles obtained are based on clinical breakpoints. The M. tuberculosis resistance profiles were detected by phenotypic drug susceptibility testing (DST), which yields MICs (22, 24). All of the interpretations were already performed based on the World Health Organization (WHO)-defined critical concentrations and are available on the Relational Sequencing TB Data Platform. The E. coli, S. aureus, and S. enterica resistance profiles were obtained by considering the available interpretations and MICs. MIC values were interpreted, based on the clinical breakpoints for resistance established by the Clinical and Laboratory Standards Institute (CLSI) for E. coli and S. aureus (25), to be consistent with the interpretations already performed for PATRIC, which were based on CLSI breakpoints. The clinical breakpoints from the European Committee on Antimicrobial Susceptibility Testing (EUCAST; http://www.eucast.org/clinical_breakpoints/) were used for S. enterica due to the limited number of resistance phenotypes observed when the CLSI breakpoints were applied. Among the interpretations already available for S. enterica, only the isolates with resistance profiles were included, as they were considered resistant according to both the CLSI and the EUCAST breakpoints.

In total, 1,785 M. tuberculosis isolates had resistance profiles for rifampin, isoniazid, streptomycin, ethambutol, and pyrazinamide and 92 M. tuberculosis isolates had complete resistance profiles for those five antimicrobials and ciprofloxacin. For the remaining M. tuberculosis isolates, the profiles for some antimicrobials were missing.

For the additional species, E. coli, S. enterica, and S. aureus, among the profiles for resistance to the antimicrobials mentioned above, only the ciprofloxacin resistance profiles were available. The two available antimicrobials for S. enterica and S. aureus (streptomycin and rifampin, respectively) needed to be excluded due to unknown breakpoints for streptomycin and an insufficient number (<5%) of resistant isolates for rifampin.

The data sets for all of the species were imbalanced, where the greatest imbalance was for ciprofloxacin in S. enterica, 5% of the isolates of which were resistant, and ethambutol and pyrazinamide in M. tuberculosis, 15% and 10% of the isolates of which, respectively, were resistant. The total number of isolates, including their phenotypes and the clinical breakpoints, used are shown in Table S1.

10.1128/mSystems.00774-19.3 TABLE S1 The total number of isolates, including their phenotype and the clinical breakpoints used. Download Table S1, DOCX file, 0.02 MB. Copyright © 2020 Aytan-Aktug et al. 2020 Aytan-Aktug et al. This content is distributed under the terms of the Creative Commons Attribution 4.0 International license.

**FIG 3:**
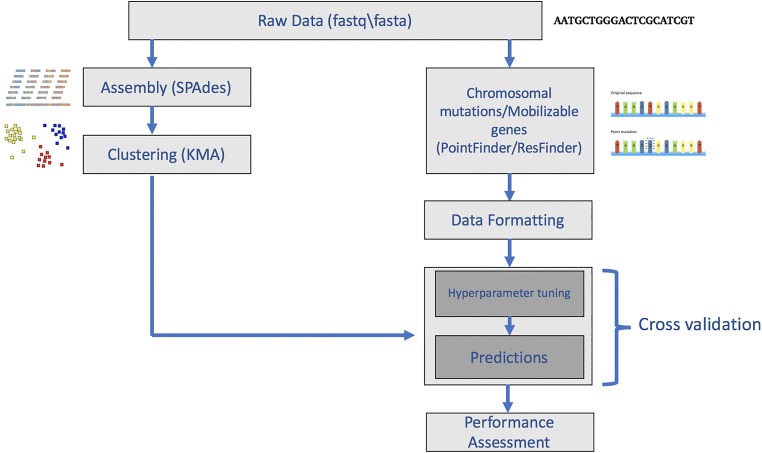
Workflow of the study. Isolates were clustered before division into training, validation, and test sets. The presence and absence of resistance-related mobilizable genes were found using the ResFinder program, and mutations in resistance-related genes were found using the PointFinder program. The output from these programs was then reformatted to be used as the input for machine learning.

The sequence data were assembled using the SPAdes algorithm (26) and were clustered using the KMA alignment method (27). These clusters were then used for machine learning. The overview of the study methodology is shown in Fig. 3.

### Data clustering.

To avoid having similar data in different training/validation/test sets, the data were clustered before being divided into sets, and the isolates in each cluster were kept together in the training/validation/test set.

Isolates were clustered based on genome similarities. Raw data in fastq/fasta format were assembled using the SPAdes algorithm (version 3.9.0) with the following parameters: -k 21, –careful, –only-assembler, and -t 16. The resulting scaffolds were merged with 16 N-nucleotide spacers in order to be treated by the clustering program. These merged scaffold files were then clustered using the KMA alignment method (version 1.1.0) with the following parameters: -k 16, -Sparse, -ht 0.9, and -hq 0.9 -NI, where the -ht and -hq parameters correspond to the template and query coverage thresholds, respectively. The values of both of the parameters were set to 0.9, corresponding to a 90% template and query coverage threshold in *k*-mer space. It should be noted that the N-nucleotide patches merging the scaffolds ensure that *k*-mers spanning more than one contig are avoided.

### PointFinder.

The PointFinder program identifies chromosomal mutations caused by point mutations and insertion/deletions (indels), while taking frameshifts, premature stop codons, RNA mutations, and promoter mutations into consideration. The PointFinder program (version 2.0) (6) was used for the detection of chromosomal mutations, not necessarily those mediating AMR, in M. tuberculosis, S. enterica, E. coli, and S. aureus. In this study, the prediction step was completed using the machine learning algorithms (see details below) instead of using prior knowledge. PointFinder was used with the KMA alignment option. For the assembled isolates, the default KMA options were changed manually in the PointFinder script. Among the default parameters, the -1t1 flag parameter was removed. The predictions made by PointFinder were compared with the machine learning prediction results. It should be noted that at the time of testing, the PointFinder database did not contain any information regarding ciprofloxacin resistance for S. aureus.

### ResFinder.

The ResFinder program (18) predicts AMR profiles based on the detection of mobilizable genes. The ResFinder tool (version 4.0) applies the KMA alignment method for detecting AMR genes in the fastq files as the default. The AMR genes detected by the KMA alignment method using the ResFinder database (version 4.0) were used as the input to the machine learning methods. The machine learning prediction results were compared with the ResFinder results produced using the default parameters.

### Data formatting.

All of the chromosomal mutations and mobilizable genes detected were merged and reformatted. The data were formatted so that the rows represent the isolates, while the columns contain information about chromosomal mutations and mobilizable genes. Basically, each feature in each column corresponds to a chromosomal mutation at some position or a mobilizable AMR gene. The representations of these features are discussed in the following subsections.

### (i) Input representation methods.

Four different input representation methods were compared.

*(a) Binary representation.* If a mutation was detected, the position was marked 1; if not, it was marked −1. Insertions and deletions were considered one position regardless of the indel size. The presence and absence of mobilizable genes were represented by the template coverages of the genes, which were repeated for the following methods.

*(b) Scored representation.* Amino acids were represented as the Blosum 62 matrix score (28) between the observed and the wild-type amino acid. Mutations in RNA genes or promoter regions were scored by use of the nucleotide substitution matrix. The nucleotide substitution matrix scores mutations as −3 and nucleotide matches as 1, which match the penalties applied by the KMA alignment method. Insertions were considered to be one position regardless of the indel size, and deletions were considered position by position. They were scored with a linear gap penalty of −5.

*(c) Amino acid representation.* In the amino acid representation, each chromosomal mutation was represented binarily by the 20 amino acids, insertions, deletions, and additional PointFinder terminology options (explained below). Furthermore, due to mutations in promoter regions or RNA genes, four nucleotide options were also added to the mentioned features to represent nucleotide mutations in noncoding regions. The feature corresponding to the observed amino acid or nucleotide was marked 1; other features were marked −1. Indels were represented as explained above for the scored representation method.

*(d) Nucleotide representation.* The nucleotide representation method shared all the properties with the amino acid representation method, except that all mutations were represented by the four nucleotide, insertions, deletions, and additional PointFinder terminology options (explained below).

**FIG 4:**
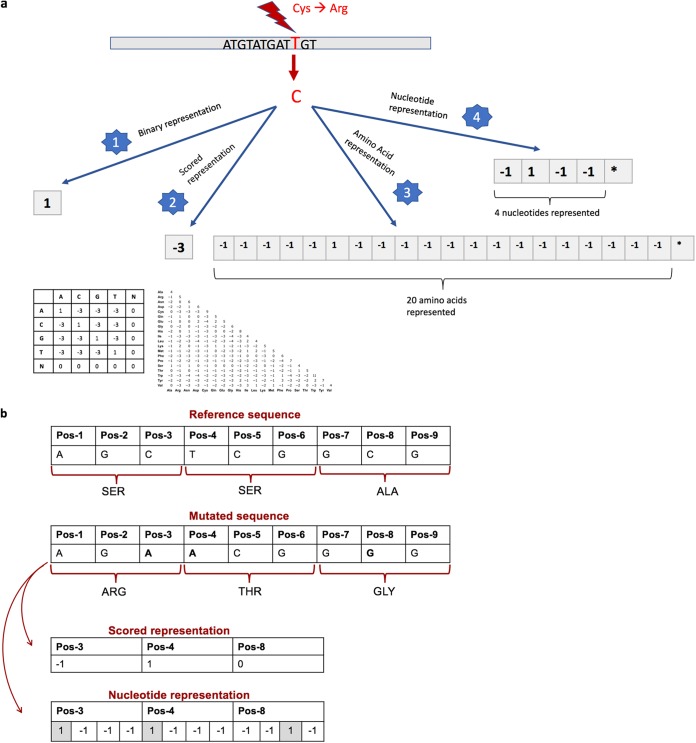
(a) Schematic representation of the four input representations tested. As demonstrated above, a thymine-to-cytosine mutation in the DNA sequence, which corresponds to a cysteine-to-arginine amino acid mutation in the protein sequence, was represented in four different ways. The binary representation method marked this as position 1, since a mutation occurred in that position. The scored representation method assigned a score of −3 to the amino acid mutation, based on the Blosum 62 matrix. The amino acid and nucleotide representation methods represented the mutation using the sparse coding, where all the possible features were represented, the corresponding feature to the mutation was marked 1; the remaining features were marked −1. *, additional features from PointFinder and insertion and deletion options. (b) An example of scored and nucleotide representation methods. Pos, position.

As mentioned above, some additional features had to be added to the amino acid and nucleotide features due to the PointFinder terminology. For instance, PointFinder depicts a stop codon, frame-restoring mutations, and unknown RNA mutations by specific characters. These extra characters are considered mutations in the binary representation method, the mutations (except for the unknown RNA mutations) are scored −5 in the scored representation method, and the characters are added to the amino acid and nucleotide representation methods as additional features. The unknown RNA mutations are scored 0 in the scored method because of the lack of information regarding the mutated version of nucleotides. The four representation methods and an example are shown in Fig. 4.

In addition, a combination of the representation methods was also tested. The binary and scored approaches were combined so that each mutation is represented by two features, the first of which includes the mutation score and the second of which has binary information regarding the absence/presence of the mutation. Furthermore, the mobilizable genes were represented again by template coverages.

### (ii) Input merging.

The following two different methods were used to create input vectors for more than one species.

*(a) Method 1.* The input matrix to the machine learning methods was separated into sections corresponding to each species. Mutations were found by running the sequence of each species against the sequences of genes known to be relevant for that species using PointFinder. All other inputs were put to −1.

*(b) Method 2.* Instead of aligning the sequence of each species to its own specific sequence database, the sequence of each species was aligned to the sequences in a combined reference database. This combined reference database includes the databases for the four different species used in the study, namely, M. tuberculosis, E. coli, S. enterica, and S. aureus. The alignment process was completed by the use of KMA via PointFinder, as in method 1. With this approach, the feature merging step was automatically included.

### (iii) Output formatting.

The AMR profiles were represented in binary format, where resistance was marked 1 and susceptibility was marked 0, according to the results of phenotypic susceptibility tests. Moreover, intermediate resistant isolates were considered resistant for M. tuberculosis. No intermediate resistant isolates occurred in the remaining data sets.

### (iv) Output merging.

The AMR profiles were merged for multioutput prediction purposes. The columns were concatenated, while the data structure was maintained as rows for isolates and columns for AMR profiles. Unavailable resistance profiles were assigned a value of −1.

### Prediction models.

AMR profiles were predicted using two different models, namely, the random forest (29) and artificial neural network (30) models.

The random forest models were constructed using the Python (version 2.7.14) package Scikit-learn (version 0.20.3) (31), with the number of trees in the forest being set to 200; the remaining parameters were left at the default settings. These parameters were determined to be optimal for all four of the representation methods.

The fully connected neural network models were constructed using the Python package PyTorch (version 1.1.0) (32). The model architectures include one to three hidden layers. The one-hidden-layer model including 200 hidden neurons was applied to all the data sets generated. The multiple-hidden-layer models were applied only to the multispecies data since this data set might require more complex models. A rectified linear unit (ReLU) was used as the activation function; in the output layer, the sigmoid activation function was applied. The stochastic gradient descent algorithm with *L*_2_ regularization was selected to update the initialized weights, and the binary cross entropy loss function was applied to calculate the cost. The learning rate was varied between the models based on the input data type. The learning rate was set to 0.01 for the binary data sets (the binary, amino acid, and nucleotide data representation methods) and to 0.001 for the continuous data set (the scored data and the combination of the binary and scored data representation methods).

The model was fed by batch inputs with a batch size of 100. The number of epochs was set to 5,000 for the multispecies data sets and 1,000 for the remaining data sets. The hyperparameters were tuned based on the average of the validation data performances. The test data were used to assess overfitting and were not utilized for hyperparameter tuning. The prediction models were applied both to the normalized (zero mean and unit variance) and to the nonnormalized data sets. The best model performances obtained using the tuned parameters were reported for the nonnormalized validation and test data sets.

In the S. enterica case, the isolates with a resistance phenotype were up-sampled due to the vastly imbalanced data set. Each resistant isolate was repeated five times in the data set, and all the repeated isolates were kept in the same cross-validation partitions.

### Cross-validation.

A 5-fold cross-validation was used for hyperparameter tuning and model assessment. The data set was divided into five partitions; one out of the five partitions was used as the validation data, and the rest were used as the training data. The final performance of the model corresponds to the average of the five performances.

**FIG 5:**
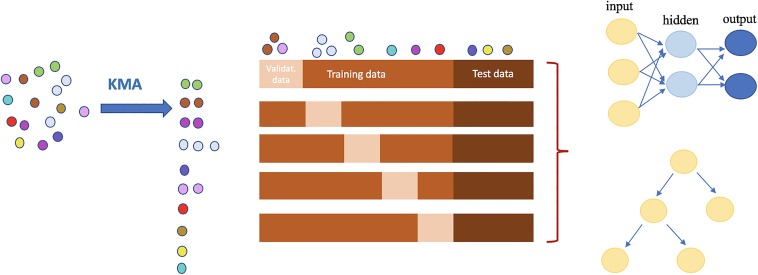
Overview of prediction strategy. First, all of the isolates are clustered on the basis of genome similarities, performed by use of the KMA method. All of the clusters are then randomly divided into three groups to prevent the training, validation (Validat.), or testing of the models with similar isolates. The neural network and random forest models, schematically drawn at the top and bottom of the right side of the figure, respectively, were applied to the data sets generated.

At the start of the study, one-sixth of the clusters were taken out as a test data set to assess the model, and the rest were used for the 5-fold cross-validation. The modified 5-fold cross-validation method is shown in Fig. 5.

### Performance assessment.

**(i) ROC curves.** The model performances were assessed with receiver operating characteristic (ROC) curves. The ROC curve plots true-positive rates, known as sensitivity, versus false-positive rates, known as one minus specificity, for all applicable thresholds. The area under the curve (AUC) was used to reduce the curves to a single number.

**(ii) Loss plots.** In order to track training and validation data performances, the loss plots were generated by the use of custom Python scripts. The loss plots show the average loss per epoch, which can be used to flag overfitting/underfitting problems.

**(iii) Paired *t* test.** The differences between the model performances were determined by paired *t* tests based on the performances obtained from the 5-fold cross-validation loop.

### Software availability.

The software used for this study is available on Bitbucket (https://deaytan@bitbucket.org/deaytan/). As a fixed seed was not used for the random weight initializer in neural networks, slightly different results (AUC, ∼0.01) might be experienced by the user.

10.1128/mSystems.00774-19.6 TABLE S4 The first 20 most important features demonstrated were generated by the neural network models. The feature importance was performed using the Shap (version 0.28.5) package (https://papers.nips.cc/paper/7062-a-unified-approach-to-interpreting-model-predictions). Each feature listed in the top 20 most important feature list at least three times in the 5-fold cross-validation loop. Since the M. tuberculosis isolates were tested for six different AMR profiles, only the top 10 most important features are listed due to the expanded list. The features overlapping the Point-/ResFinder databases are written in bold. S. aureus results were not comparable because the PointFinder database did not include any information regarding ciprofloxacin at the time of testing. For each mutation, the order is GeneName_Position_WildType. Del, deletion. Download Table S4, DOCX file, 0.02 MB. Copyright © 2020 Aytan-Aktug et al. 2020 Aytan-Aktug et al. This content is distributed under the terms of the Creative Commons Attribution 4.0 International license.

10.1128/mSystems.00774-19.8 DATA SET S2 The most 50 important features in the raw format for the random forest models. Download Data Set S2, XLSX file, 0.01 MB. Copyright © 2020 Aytan-Aktug et al. 2020 Aytan-Aktug et al. This content is distributed under the terms of the Creative Commons Attribution 4.0 International license.

10.1128/mSystems.00774-19.9 DATA SET S3 The most 50 important features in the raw format for the neural network models. Download Data Set S3, XLSX file, 0.01 MB. Copyright © 2020 Aytan-Aktug et al. 2020 Aytan-Aktug et al. This content is distributed under the terms of the Creative Commons Attribution 4.0 International license.
