# Supplementary material for: Prediction of Acquired Antimicrobial Resistance for Multiple Bacterial Species Using Neural Networks
Source: mSystems. 2020 Jan 21;5(1):e00774-19. doi: 10.1128/mSystems.00774-19 (PMC6977075; doi:10.1128/mSystems.00774-19)
Supplement: TABLE S3 [file mSystems.00774-19-st003.docx]

| *E. coli* | *M. tuberculosis* | *S. enterica* | *S. aureus* |
| --- | --- | --- | --- |
| *parC*_471_A | ***rpoB*_450_S** | *tet(B)_*2_AF326777 | *grlA*_650_K |
| *parE*_458_S | ***fabg1*_*promoter*_-15_C** | ***gyrA*_83_S** | *pbp4*_*promoter*_3_L |
| *parE*_529_I | ***rpoB*_445_H** | *tet(G)_*2_AF133140 | *pbp4*_395_S |
| *mph(A)* 1_D16251 | ***rrs*_1401_A** | ***oqxB*_1_EU370913** | *grlA*_404_E |
| *parC*_80_S | ***katG*_315_S** | *blaCARB-2*_1_M69058 | *grlA*_80_S |
| *gyrA*_87_D | ***rpsL*_88_K** | *mcr_9*_1_NZ_NAAN01000063.1 | *pbp4*_322_S |
| *gyrA*_83_S | *erm(37)_*1_AL123456 | ***qnrB19*_1_EU432277** | *gyrA*_84_S |
| *aac(6')-Ib-cr*_1_DQ303918 | ***gyrA*_94_D** | *23S*_60_del | *pbp2*_113_T |
| *parC*_192_A | *aac(2')-Ic*_1_U72714 | ***gyrA*_87_D** | *erm(C)_*13_M13761 |
| *parC*_481_Q | ***rpsL*_43_K** | *floR*_2_AF118107 | *grlB*_289_T |
| *parC*_84_E | ***embB*_306_M** | *ARR-3*_4_FM207631 | *ileS*_147_D |
|  |  | *16S_rrsd*_1022_G | *dfrB*_135_A |
|  |  | *sul1*_5_EU780013 | *ileS*_150_D |
|  |  | *aadA2*_1_NC_010870 | *pbp2*_557_A |
|  |  | *dfrA12*_8_AM040708 |  |
