# Supplementary figures and images for: Prediction of Acquired Antimicrobial Resistance for Multiple Bacterial Species Using Neural Networks
Source: mSystems. 2020 Jan 21;5(1):e00774-19. doi: 10.1128/mSystems.00774-19 (PMC6977075; doi:10.1128/mSystems.00774-19)

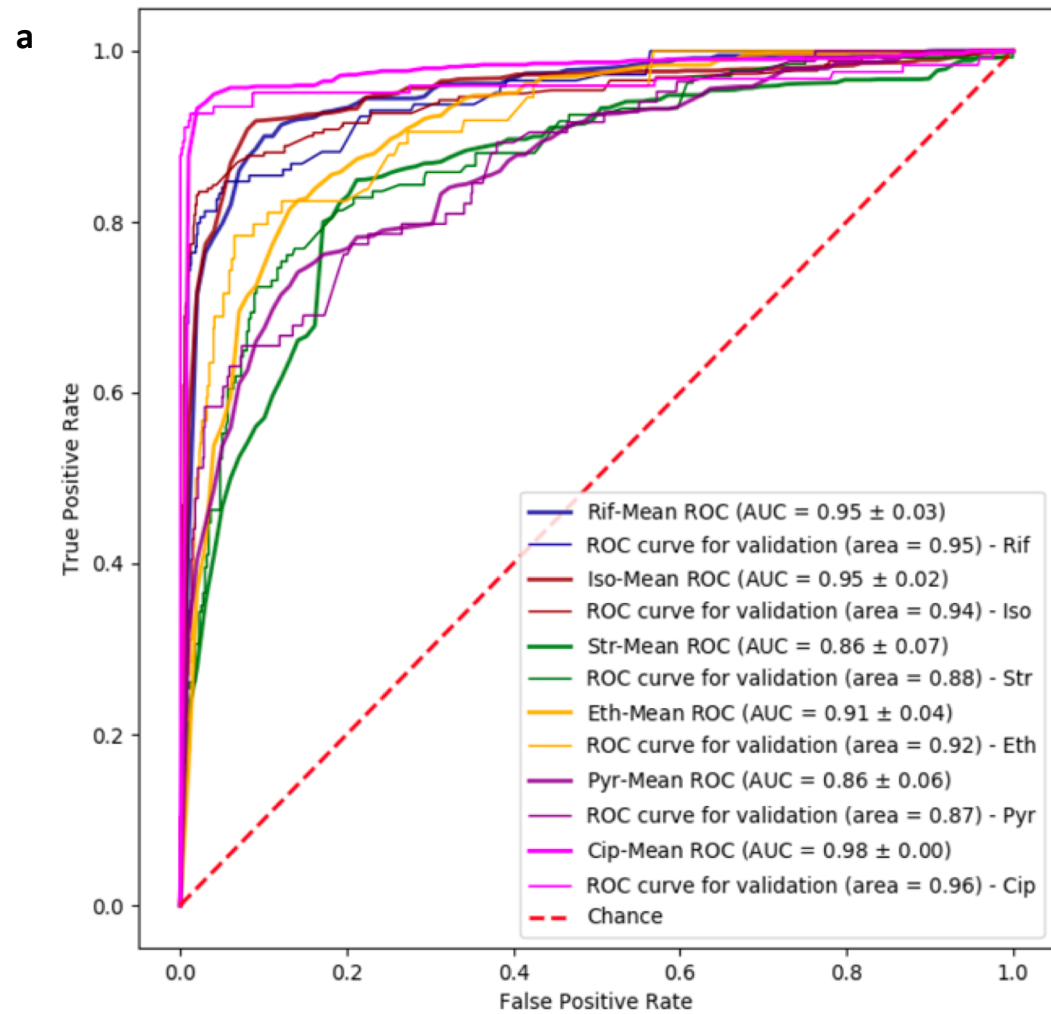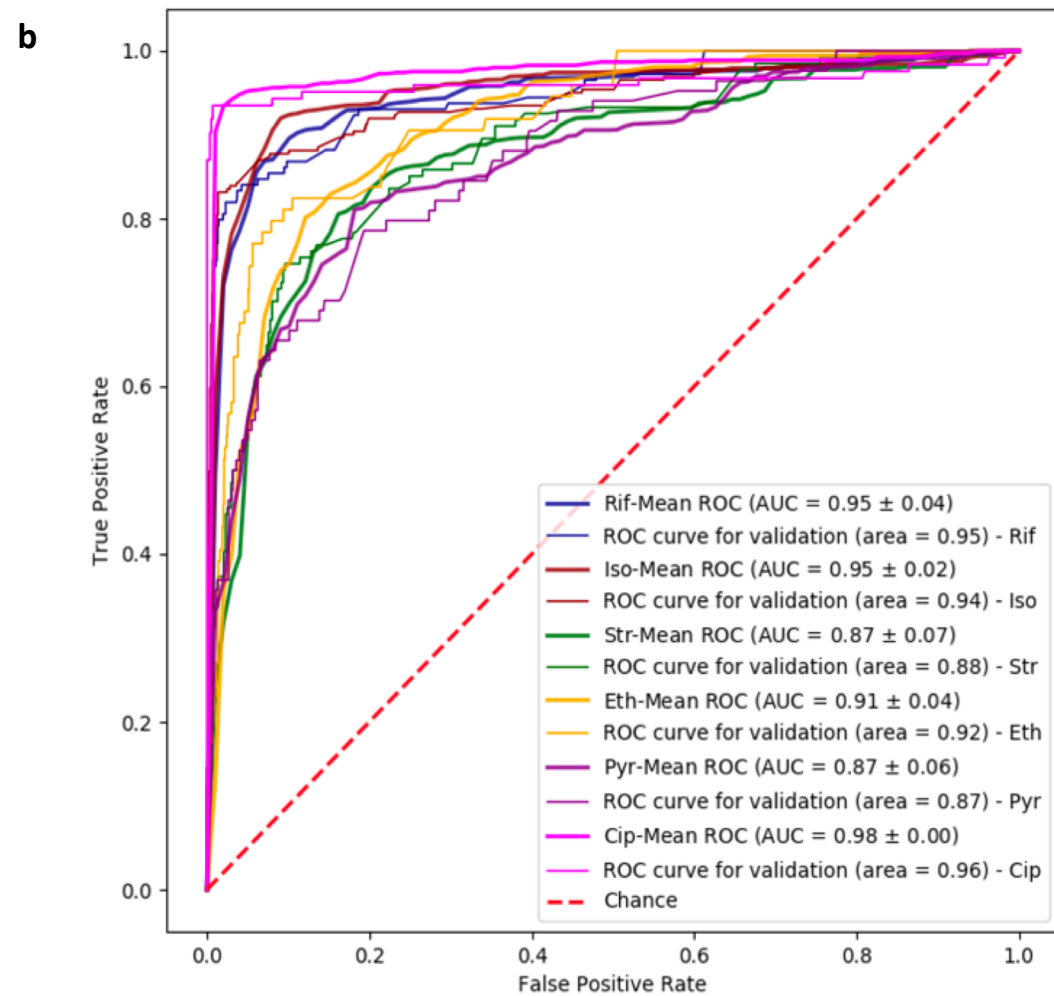

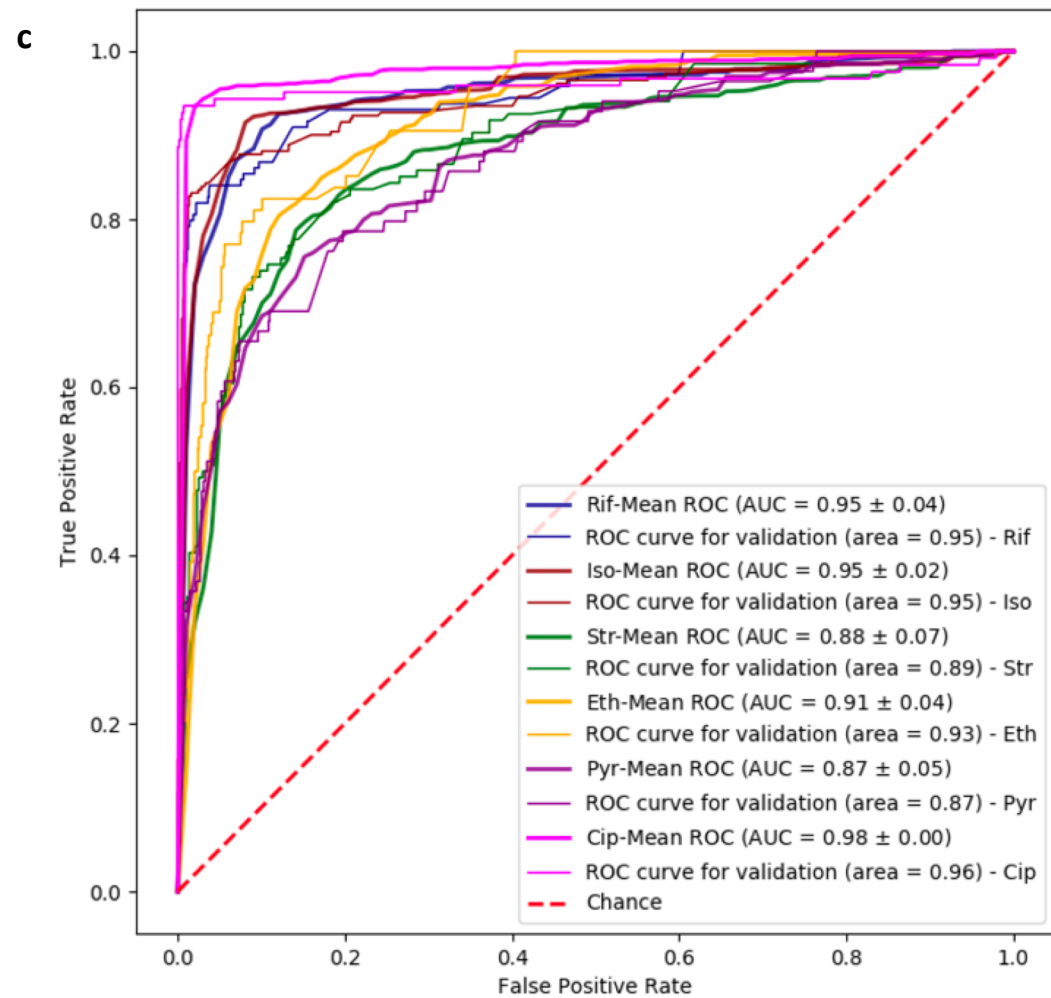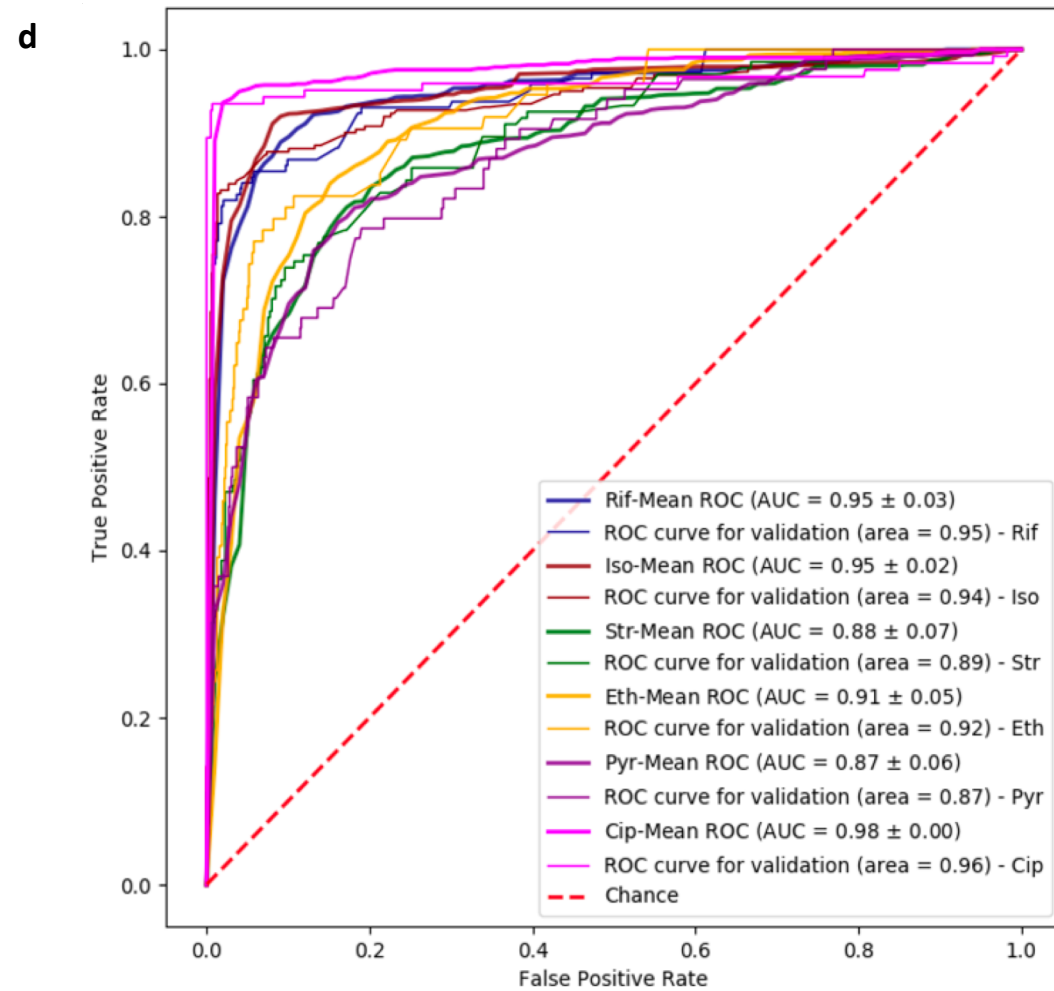

Supplement: FIG S1 [file mSystems.00774-19-sf001.pdf]

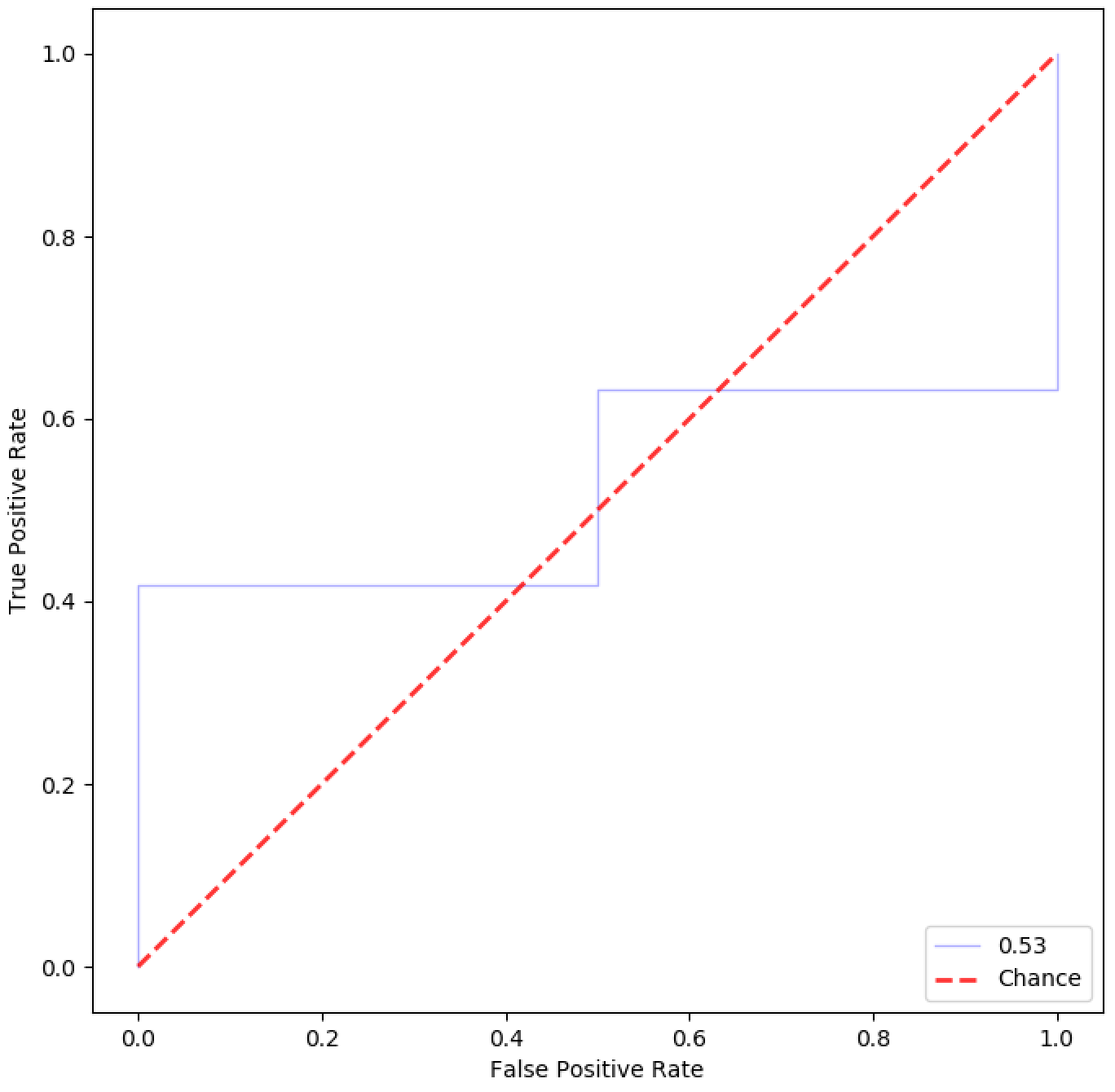

Supplement: FIG S2 [file mSystems.00774-19-sf002.tif]
