# Supplementary material for: Prediction of Acquired Antimicrobial Resistance for Multiple Bacterial Species Using Neural Networks
Source: mSystems. 2020 Jan 21;5(1):e00774-19. doi: 10.1128/mSystems.00774-19 (PMC6977075; doi:10.1128/mSystems.00774-19)
Supplement: TABLE S2 [file mSystems.00774-19-st002.docx]

| Species | Point/ResFinder | | | | | | Machine Learning | | | | | | |
| --- | --- | --- | --- | --- | --- | --- | --- | --- | --- | --- | --- | --- | --- |
|  | **Validation data** | | | **Test data** | | | **Validation data** | | | | **Test data** | | |
|  | Sensitiv. | Specif. | F-1 | Sensitiv. | Specif. | F-1 | Sensitiv. | Specif. | F-1 | Sensitiv. | | Specif. | F-1 |
| *E. coli*  /Ciprofloxacin | 0.98 | 0.85 | 0.76 | 0.97 | 0.84 | 0.75 | 0.96 | 1.0 | 0.97 | 0.95 | | 0.99 | 0.95 |
| *M. tuberculosis*  /Ciprofloxacin | 0.93 | 0.98 | 0.93 | 0.80 | 0.97 | 0.73 | 0.61 | 0.95 | 0.70 | 0.80 | | 0.98 | 0.80 |
| *M. tuberculosis*  /Rifampicin | 0.79 | 0.99 | 0.85 | 0.93 | 0.94 | 0.92 | 0.64 | 0.99 | 0.72 | 0.85 | | 0.95 | 0.88 |
| *M. tuberculosis*  /Isoniazid | 0.85 | 0.98 | 0.89 | 0.93 | 0.95 | 0.94 | 0.42 | 0.99 | 0.56 | 0.73 | | 0.96 | 0.83 |
| *M. tuberculosis*  /Streptomycin | 0.61 | 0.95 | 0.68 | 0.94 | 0.83 | 0.90 | 0.42 | 0.93 | 0.46 | 0.74 | | 0.84 | 0.79 |
| *M. tuberculosis*  /Ethambutol | 0.76 | 0.95 | 0.62 | 0.89 | 0.78 | 0.70 | 0.18 | 0.99 | 0.14 | 0.07 | | 0.99 | 0.12 |
| *M. tuberculosis*  /Pyrazinamide | 0.52 | 0.98 | 0.61 | 0.63 | 0.95 | 0.66 | 0.25 | 0.99 | 0.32 | 0.21 | | 0.97 | 0.30 |
| *S. enterica*  /Ciprofloxacin | 0.83 | 1.0 | 0.89 | 0.8 | 1.0 | 0.89 | 0.51 | 0.95 | 0.54 | 0.40 | | 0.92 | 0.47 |
| *S. aureus*/  Ciprofloxacin | NA | NA | NA | NA | NA | NA | 0.97 | 0.99 | 0.98 | 0.93 | | 1.0 | 0.97 |
