# Supplementary material for: Prediction of Acquired Antimicrobial Resistance for Multiple Bacterial Species Using Neural Networks
Source: mSystems. 2020 Jan 21;5(1):e00774-19. doi: 10.1128/mSystems.00774-19 (PMC6977075; doi:10.1128/mSystems.00774-19)
Supplement: TABLE S1 [file mSystems.00774-19-st001.docx]

| Species/Antimicrobial | Number of isolates | Number of Resistant isolates | Number of susceptible isolates | Clinical Breakpoints of Resistance based on MIC values (µg/ml) |
| --- | --- | --- | --- | --- |
| *E. coli*/Ciprofloxacin | 1,694 | 343 | 1,351 | ≥ 1 |
| *M. tuberculosis*/Ciprofloxacin | 339 | 60 | 279 | NA |
| *M. tuberculosis*/Rifampicin | 3,481 | 771 | 2,710 | NA |
| *M. tuberculosis*/Isoniazid | 3,515 | 1,095 | 2,420 | NA |
| *M. tuberculosis*/Streptomycin | 1,967 | 728 | 1,239 | NA |
| *M. tuberculosis*/Ethambutol | 3,506 | 466 | 3,040 | NA |
| *M. tuberculosis*/Pyrazinamide | 3,320 | 327 | 2,993 | NA |
| *S. enterica*/Ciprofloxacin | 658 | 35 | 623 | > 0.06 |
| *S. aureus*/Ciprofloxacin | 1,236 | 469 | 767 | ≥ 4 |
