# Supplementary material for: Prediction of Acquired Antimicrobial Resistance for Multiple Bacterial Species Using Neural Networks
Source: mSystems. 2020 Jan 21;5(1):e00774-19. doi: 10.1128/mSystems.00774-19 (PMC6977075; doi:10.1128/mSystems.00774-19)
Supplement: TABLE S4 [file mSystems.00774-19-st004.docx]

| *E. coli* | *M. tuberculosis* | *S. enterica* | *S. aureus* |
| --- | --- | --- | --- |
| *ampC*_9_L | ***katG*_463_R** | *16S_rrsd*_1022_del | *gyrA*_84_S |
| *gyrA*_87_D | ***rpoB*_450_S** | *23S*_138_T | *grlA*_80_S |
| *pmrA*_144_G | *gyrB*_94_P | ***gyrA*_87_D** | *grlA*_404_E |
| *ampC*_8_R | *rrl*_344_del | *16S_rrsd*_1022_G | *pbp2*_113_T |
| *23S*_2793_C | ***rpoB*_435_D** | *23S*_138_del | *pbp4*_322_S |
| *parC*_80_S | *gidB*_48_H |  | *grlB*_289_T |
| *parC*_84_E | ***rpoB*_445_H** |  | *gyrA*_817_E |
| *parC*_481_Q | *rrl*_344_C |  | *pbp2*_717_R |
|  | ***embB*_406_G** |  |  |
|  | ***katG*_315_S** |  |  |
|  | ***embB*_306_M** |  |  |
|  | *gidB*_16_L |  |  |
|  | *embA*_913_P |  |  |
|  | *rrs*_492_C |  |  |
|  | *embC*_394_N |  |  |
|  | *iniA*_481_H |  |  |
|  | *drrA*_309_H |  |  |
|  | *embR*_110_C |  |  |
|  | *idsA2*_*promoter*_-98_C |  |  |
|  | *embC*_567_R |  |  |
|  | ***fabg1_promoter*_-15_C** |  |  |
|  | ***embB*_378_E** |  |  |
|  | *thyA*_202_T |  |  |
|  | *gyrA*_668_G |  |  |
|  | *embC*_738_R |  |  |
|  | ***embC*_270_T** |  |  |
|  | ***rpsL*_43_K** |  |  |
|  | *ahpC_promoter*_-88_G |  |  |
|  | ***gyrA*_94_D** |  |  |
